# Insights from a Genome-Wide Study of *Pantoea agglomerans* UADEC20: A Promising Strain for Phosphate Solubilization and Exopolysaccharides Production

**DOI:** 10.3390/cimb47010056

**Published:** 2025-01-16

**Authors:** Edith Elizondo-Reyna, Humberto Martínez-Montoya, Yahaira Tamayo-Ordoñez, María Antonia Cruz-Hernández, Mauricio Carrillo-Tripp, María Concepción Tamayo-Ordoñez, Gerardo de Jesús Sosa-Santillán, José Antonio Rodríguez-de la Garza, Mario Hernández-Guzmán, Virgilio Bocanegra-García, Erika Acosta-Cruz

**Affiliations:** 1 Departamento de Biotecnología, Facultad de Ciencias Químicas, Universidad Autónoma de Coahuila, Saltillo C.P. 25280, Mexico; 2 Departamento de Microbiología, U.A.M. Reynosa Aztlán, Universidad Autónoma de Tamaulipas, Reynosa C.P. 88740, Mexico; 3 Laboratorio Interacción Ambiente-Microorganismo, Centro de Biotecnología Genómica, Instituto Politécnico Nacional, Reynosa C.P. 88710, Mexico; 4 Biomolecular Diversity Laboratory, Centro de Investigación y de Estudios Avanzados del Instituto Politécnico Nacional, Unidad Monterrey, Vía del Conocimiento 201, PIIT, Apodaca C.P. 66600, Mexico; 5 Departamento de Innovación Biomédica, Centro de Investigación Científica y de Educación Superior de Ensenada (CICESE), Ensenada C.P. 22860, Mexico

**Keywords:** PGPR, genome, *Pantoea agglomerans*, phosphate solubilization, exopolysaccharide production

## Abstract

The genome sequence of *Pantoea agglomerans* UADEC20 is presented, which is a strain isolated from agricultural fields in northeast Mexico. The genome was assembled into 13 scaffolds, constituting a total chromosome size of 4.2 Mbp, with two of the scaffolds representing closed plasmids. The strain exhibits activity in phosphate solubilization and exopolysaccharide (EPS) production and secretion; therefore, we explored its biotechnological potential via its genome sequencing and annotation. Genomic analyses showed that a total of 57 and 58 coding sequences (CDSs) related to phosphate solubilization and EPS production were identified within its genome, in addition to a reduced number of CDSs related to drug resistance and phages. The comprehensive set of genes supporting phosphate solubilization, EPS synthesis, and secretion, along with its low virulence and antibiotic resistance levels, justify further research for its potential biotechnological application and possible use as a plant growth-promoting agent in the field. These findings suggest a unique genetic background in the *P*. *agglomerans* UADEC20 strain.

## 1. Introduction

*Pantoea agglomerans* is a Gram-negative, non-encapsulated, non-sporulated bacillus, belonging to the Enterobacteriaceae family, which is included in the class of Gammaproteobacteria that includes facultative anaerobic and fermentative Gram-negative bacteria. *P. agglomerans* is the most studied species and model of the genus *Pantoea*, which includes approximately 20 species [1,2]. The wide biotechnological applications of this species have led the scientific community to focus on studying microbial cultures associated with important agricultural crops in Mexico. *Pantoea* spp. are known to enhance plant growth by increasing the nitrogen supply, solubilizing ammonia and inorganic phosphate, and producing phytohormones [1,3,4]. *P. agglomerans* exhibits great adaptability to a wide range of hosts, diverse environmental conditions, and geographic and ecological distributions. This adaptability positions this bacterium as an opportunity for use as a biological control agent and a biofertilizer, with plant growth-promoting activity (PGPR) and potential use for soil bioremediation. The known mechanisms of PGPRs are diverse, e.g., nitrogen fixation, phytohormone production, and phosphate solubilization [5,6,7,8,9].

For all the above properties, the production of acid phosphatases and organic acids, particularly gluconic acid (GA), is vital for solubilizing insoluble or poorly soluble mineral phosphates [10]. Moreover, exopolysaccharide (EPS) production has an important role in plant growth and drought tolerance [11]. Previous studies have focused on the sequencing of the strains of *Pantoea* species to investigate the genes and enzymes involved in direct and indirect genetic mechanisms of plant growth-promoting bacteria [12,13,14]. When using PGPRs for growth enhancement, it is preferable to use locally isolated strains since they have evolved to adapt to the chemical, physical, and biological soil conditions, which enhances their biotechnological potential. The genomic data on local strains such as UADEC20 and its properties help to acquire a better understanding of the physiology, adaptability, and potential use of these strains.

The aim of this study was the genomic characterization of a strain that was isolated from the alfalfa (*Medicago sativa*) rhizosphere with technified irrigation in northeast Mexico. The analysis included the sequencing, assembly, annotation, and genomic, structural, and functional characterization of *P. agglomerans* UADEC20. This genome presents important properties such as phosphate solubilization and EPS production, which renders it a promising candidate for its usage in agriculture and, therefore, for enhancing its further biotechnological applications.

## 2. Materials and Methods

### 2.1. Whole-Genome Sequencing, Assembly, and Annotation

The strain *P. agglomerans* UADEC20 was isolated from the rhizosphere of alfalfa plants (*Medicago sativa*) cultivated in soil with a pH of 8.1 in the northeastern region of Mexico (25°1′40″ N, 100°38′28″ W). After several serial dilutions and plating on PVK medium at 28 ℃, it was identified as *P. agglomerans* through 16S rDNA sequencing and analysis with BLAST [15]. Total DNA extraction and purification were performed using the Wizard Genomic DNA Purification Kit (Promega Corporation, Madison, WI, USA) following the manufacturer’s recommendations. DNA integrity analysis to determine its feasibility for next-generation sequencing was performed on an Agilent 2200 TapeStation System from Agilent Technologies. Whole-genome sequencing was carried out by the company Omega Bioservices (Norcross, GA, USA) using the Illumina HiSeq™ sequencing platform (2 × 150 paired-end, PE).

The quality of the raw reads was verified with FastQC v0.11.9 [16]. Trim Galore v0.6.6 (https://www.bioinformatics.babraham.ac.uk/projects/trim_galore/ [accessed on 15 April 2024]) was used to trim reads with Q-scores < 30, remove adapters, and discard reads < 50 bp. Genome assembly was carried out using the QIAGEN CLC Genomics Workbench bioinformatics software v10.0 (QIAGEN, Aarhus, Denmark). Assembly quality was assessed further with QUAST v5.0.2 [17], and genome integrity assessment was performed using BUSCO v4.0.5 [18]. The BUSCO tool employs quantitative metrics to assess genome assembly, using evolutionary information [18].

### 2.2. Phylogenetic Analysis

A phylogenetic analysis of *P. agglomerans* was conducted by aligning the 16S rRNA sequence from our strain with selected *Pantoea* species and strains retrieved from the data mining of previously sequenced strains (NCBI). The alignment was finalized using MUSCLE v3.7 via the CIPRES platform [19]. The final alignment was cured to eliminate ambiguous sequences and gaps using Gblocks 0.91b [20]. Phylogenetic reconstruction was performed using a maximum likelihood approach via the IQ-TREE server [21] (http://www.iqtree.org/ [accessed on 10 September 2024]), calculating the evolution model with ModelFinder [22]. The taxa used in the phylogenetic reconstruction were the *P. agglomerans* strains FDAARGOS 1447 (GenBank acc. no. NZ_CP077368), UADEC20 (CP125809), NCTC9381 (OQ619142), DSM 3493 (KY013009), NBRC 102470 (NR_114111), 33.1 (CP0830.1), L15 (CP034148.1), UAE018 (CP048033.1), DAPP-PG734 (OW970315.1), J22C (CP162620.1), PSV1-7 (CP091189.1), AR1a (CP059089.1), Y03 (CP144368.1), and AB378 (CP113085.1). In addition to the *P. agglomerans* strains, the following were included: *P. ananatis* strains 1846 (GenBank acc. no. NR_02604) and LMG 2665 (NR_119362), *P. allii* strain LMG 24248 (NZ_NTMH00000000), *P. stewartii* subsp. *indologenes* strain CIP 104006 (NR_104928), *P. pleuroti* strain JZB 2120015 (NR_178694), *P. vagans* strain LMG 24199 (NZ_CP038855), and *P. rwandensis* strain ND04 (CP009454.1).

### 2.3. Comparative Genome Annotation

Genome annotation was performed with the Prokaryotic Rapid Genome Annotation algorithm (Prokka v1.13; https://github.com/tseemann/prokka, accessed on 10 September 2024) [23]. The resulting *P. agglomerans* UADEC20 scaffolds were rearranged and aligned using the Mauve Contig Mover algorithm in Mauve (v2.4.0) [24] and aligned against the complete genome of *P. agglomerans* FDAARGOS1447 as a reference (GenBank accession number: CP077366.1). The two plasmids detected in *P. agglomerans* UADEC20 were aligned with the respective plasmids from *P. agglomerans* FDAARGOS1447 using Mauve [24]. In addition to Mauve, an alignment of the *P. agglomerans* UADEC20 genome against other strains of *P. agglomerans* was conducted. A Venn diagram of the pan-genome and core-genome of *P. agglomerans* UADEC20 with other strains was made, using the MicroScope (MaGe) platform of Genoscope´s bioinformatics resources [25]. A pairwise genomic alignment of UADEC20 with 6 closely related *P. agglomerans* strain genomes was plotted using NCBI databases with BRIG 0.95 [26]. The same strains were compared for genomic features including genome size, gene number, number of tRNAs and rRNAs, and GC content.

### 2.4. Predictions of Functional Metabolic Pathways

The complete genome of *P. agglomerans* UADEC20 was subjected to metabolic pathway predictions using Gapseq v1.2 [27]. The reconstruction of metabolic networks consists of the systematic annotation of genomic metabolic genes that are then linked to the corresponding enzymatic reactions. This program was used to predict the routes and transporters with the Gapseq find script (-b 150), and the network project was created using the Gapseq draft (-u 200 -l 100 -a 1). For the transporter search, sequence data from the Transporter Classification Database (TCDB) [28] were used. The set of sequences was reduced to a subset of transporters involving metabolites known to be produced or consumed by microorganisms (dat/sub2pwy.csv, accessed on 10 September 2024). It was compared with relevant pathways available in the Kyoto Encyclopedia of Genes and Genomes (KEGG) pathway database (https://www.genome.jp/kegg/pathway.html, accessed on 10 September 2024).

The visualization of metabolic pathways’ prediction was achieved as follows [29,30]: the generic feature annotation file (GFF Prokka’s annotation output) was filtered to retain all genes with a KO identifier assigned. Then, the “Minimal set of Pathways” tool (MinPath v1.4) [31] was used to obtain a conservative estimation of pathways to visually represent them on the microbial metabolic map through the interactive pathway explorer web-based tool (iPath v3, https://pathways.embl.de/ (accessed 5 October 2024)).

### 2.5. Virulence Factors

The BLASTn method was applied to the Virulence Factor Database (VFDB) [32] using an identity limit of 80% and an e-value of <10^−6^ (BLAST) to find virulence factor genes present in the *P. agglomerans* UADEC20 genome.

### 2.6. Prophages and Drug Resistance Genes

In silico identification of antibiotic resistance genes using the CARD (Comprehensive Antibiotic Resistance Database) and the Resistance Gene Identifier (RGI) was performed [33]. To support rapid identification, annotation, and visualization of prophage sequences within the bacterial genomes and plasmids, the PHASTEST (PHAge Search Tool with Enhanced Sequence Translation) web server was used [34].

## 3. Results

### 3.1. Whole-Genome Sequencing, Assembly, and Annotation

A total of 20,949,701 paired-end raw reads were obtained in this study. After trimming and QC filtering, a total of 20,873,634 bp reads were retained and used for downstream analysis. The de novo assembly of the clean reads resulted in 15 scaffolds with an N50 value of 209,743 bp. The longest scaffold was 1,523,742 bp in length. Thirteen assembled scaffolds corresponded to the chromosome, while the other two were identified as plasmids, i.e., plasmid 1 (CP125810), with a length of 543,479 bp and G+C content of 53.6%, and plasmid 2 (CP125811), with a length of 174,380 bp and G+C content of 52.0%. *P. agglomerans* UADEC20 has a single circular chromosome of 4,203,428 bp in length, with a G+C content of 54.94% (Table 1). The evaluation of genome completeness using BUSCO revealed that *P. agglomerans* UADEC20 was 99.1% complete, suggesting that most of the recovered genes could be classified as complete and single copies (Figure S1).

### 3.2. Phylogenetic Analysis

**Figure 1:**
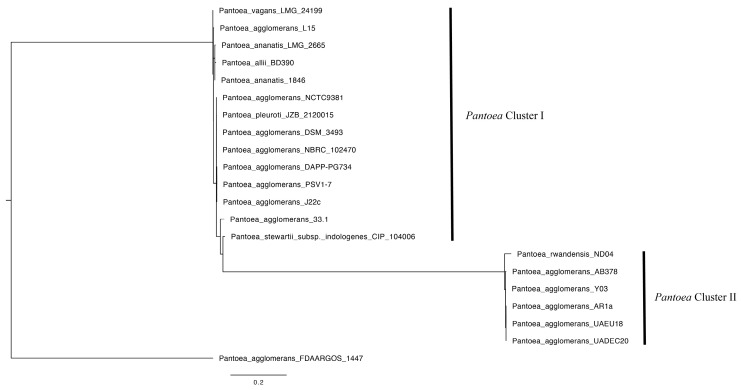
Consensus tree showing the phylogenetic relationship of 21 strains from *Pantoea.* The final tree was constructed from 2000 bootstrap trees. *Pantoea agglomerans* UADEC20 is located in Cluster II.

A total of 21 sequences were used to assess the phylogenetic reconstruction of the 16S rRNA gene. After curating the sequences in Gblocks, 620 bases were retained, representing 29% of the original alignment with 2082 nucleotide positions. The cured alignment underwent a maximum likelihood analysis to estimate the phylogenetic relationships. It contained 184 constant sites (29.6%) and 286 parsimony informative sites. The best evolution model according to the Bayesian information criterion (BIC) (5640.8911) was K2P+G4. The consensus tree shows two main clades when rooted to *P. agglomerans* FDARGOS 1447 (FIG) and suggests a close relationship with *P. agglomerans* strains UAEU18, AR1a, Y03, AB378, and ND04 in a cluster composed only of *P. agglomerans* strains (Figure 1).

### 3.3. Comparative Genome Annotation

**Figure 2:**
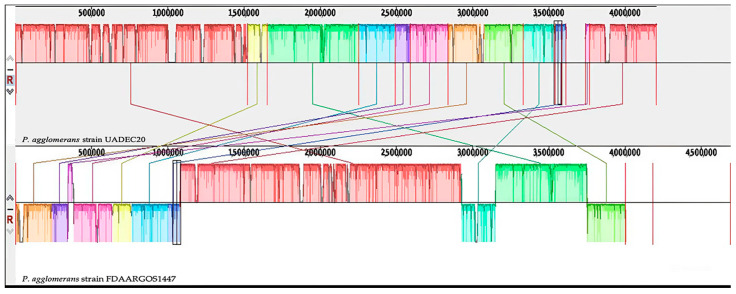
Genome alignments showing synteny blocks of *P. agglomerans* UADEC20 (**top**) and *P. agglomerans* FDAARGOS 1447 (**bottom**) in Mauve 2.4.0 software. Each genome is presented horizontally with the homologous segment outlined as colored rectangles. Blocks of the same color represent a locally collinear block (LCB) or homologous region shared between the two genomes. The rearrangement of the genomic regions between the two genomes was observed in terms of collinearity; the regions inverted relative to FDAARGOS 1447 are located on the negative strand indicated by the genomic position below the black horizontal center line in the Mauve alignment. Colors indicates similar nucleotide regions between the two chromosomes.

Annotation of the *P. agglomerans* UADEC20 chromosome with Prokka [23] revealed that a total of 4511 genes were predicted: 3836 coding sequences (CDSs), 1 tmRNA, 22 rRNA operons, 99 tRNA genes, and 716 hypothetical proteins. Functional annotation allowed the identification of 1617 enzymes (EC number of annotated genes) that were visualized through the KEGG’s microbial metabolism map (Figure S2). The *P. agglomerans* UADEC20 genome was compared with different species and strains, with the *P. agglomerans* FDAARGOS 1447 strain (GenBank accession no. CP077366.1) being the tree root. The similarity can be observed in the results of the analysis conducted with the progressive software Mauve, which builds multiple genomic alignments in the presence of evolutionary events such as rearrangements and inversions. The chromosomal alignment between the two strains revealed the presence of 12 highly homologous blocks. However, a region in the chromosomal scaffold between scaffolds 3, 8, and 10 exhibits an inverse orientation, indicating differences in their synteny ratios (Figure 2). Furthermore, strain UADEC20 possesses a total of 49 unique coding sequences (CDSs) that are absent in strain FDAARGOS 1447, as outlined in (Table S1).

**Figure 3:**
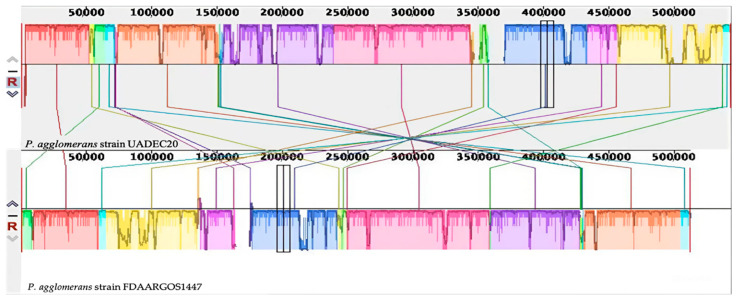
Plasmid 1 of *P. agglomerans* UADEC20 (**top**) and plasmid 1 of *P. agglomerans* FDAARGOS 1447 (**bottom**) compared using Mauve 2.4.0 software. Colors indicates similar nucleotide regions between the two chromosomes.

**Figure 4:**
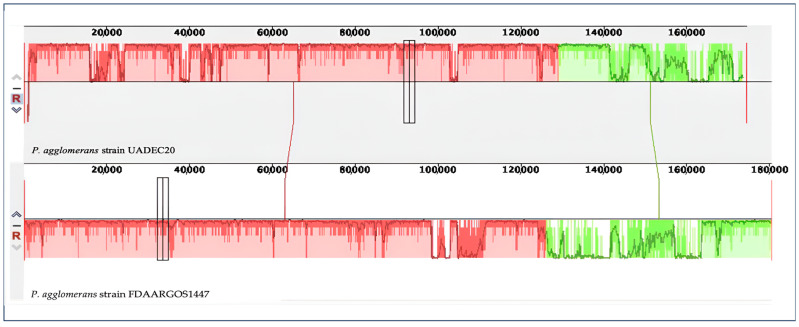
Plasmid 2 of *P. agglomerans* UADEC20 (**top**) and plasmid 2 of *P. agglomerans* FDAARGOS 1447 (**bottom**) compared using Mauve 2.4.0 software. Colors indicates similar nucleotide regions between the two chromosomes.

In the annotation of the plasmids, through an analysis with Prokka [23], a plasmid 1 (GenBank accession no. CP125810), with a size of 543,479 bp, containing 517 putative coding sequences, and a plasmid 2 (GenBank accession no. CP125811), with a size of 174,380 bp, containing 162 putative coding sequences, were predicted. The two plasmids contained in *P. agglomerans* UADEC20 were compared with the two plasmids of the *P. agglomerans* FDAARGOS 144 strain using progressiveMauve (v2.4.0), presenting two collinear blocks for each plasmid (Figure 3 and Figure 4).

**Figure 5:**
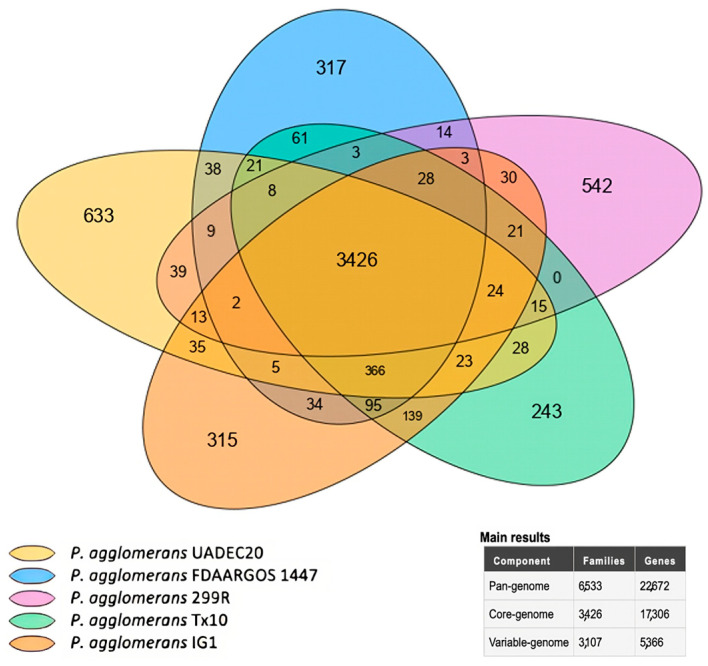
Plot of the core-/pan-genome and Venn diagram for the core-genome and strain-specific CDSs of the *P. agglomerans* strains. The outer circles represent the pan-genomes of the different strains and show the conserved (core) and non-conserved (flexible) CDSs for each group. Genes overlapping by at least 80% in length and having 80% similarity were considered orthologs.

In an overall analysis, the pan-genome is made up of 22,672 genes belonging to 6533 families, the core-genome is made up of 17,306 genes belonging to 3426 families, and the variable-genome is made up of 5366 genes belonging to 3107 families. Genes overlapping by at least 80% in length and having 80% similarity were considered orthologs (Figure 5).

**Figure 6:**
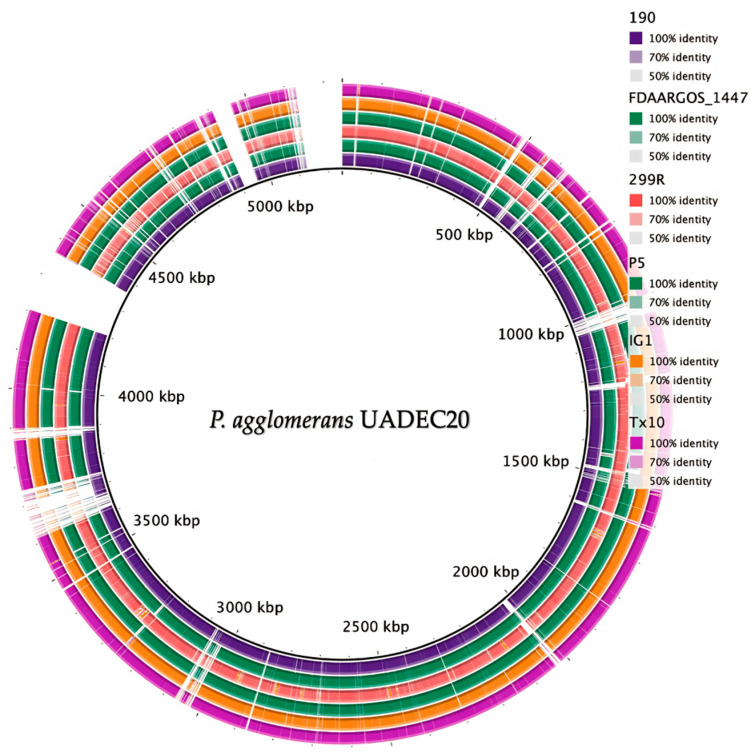
Genome comparisons of other *P. agglomerans* strains against the *P*. *agglomerans* UADEC20 draft genome. The black inner circle represents the complete genome of the reference strain UADEC20, and the shade of each color shows the similarities between each one of the strains and that of UADEC20.

Additionally, the genomic features of *P. agglomerans* strain UADEC20 and six closely related genomes were compared, such as sequence similarity, GC content distribution, and gene number, including tRNA and rRNA (Figure 6, Table 2). The genome size and total gene number of UADEC20 are similar to 299R but slightly smaller than those of FDAARGOS1447 and IG1, while 190, Tx10, and P5 appear to have a larger genome size of ~4.85 Mb.

### 3.4. Predictions of Functional Metabolic Pathways

#### 3.4.1. Predictions of Metabolic Pathways Related to Phosphate Solubilization

After annotation of the chromosome of *P. agglomerans* UADEC20, 57 CDSs related to phosphate solubilization were identified using tools such as Prokka [23] and the UniProt database (https://www.uniprot.org/, accessed on 10 September 2024). These identified CDS were completely found within the chromosome (Table 3). 

In the analysis of the predictions of the metabolic pathways obtained from the *P. agglomerans* UADEC20 genome related to the phosphate solubilization mechanism (Figure S2), using the Gapseq program (KEGG database), the following were found: D-gluconate degradation (GLUCONSUPER-PWY), glucose and glucose-1-phosphate degradation (GLUCOSE1PMETAB-PWY), sedoheptulose bisphosphate bypass (PWY0-1517), glycerol-3-phosphate to cytochrome *bo* oxidase electron transfer (PWY0-1561), succinate to cytochrome bd oxidase electron transfer (PWY0-1353), glycerol-3-phosphate shuttle (PWY-6118), phosphate acquisition (PWY-6348), and phosphate ABC transporter (PI-ABC-TRANS). In relation to the inference of transporters in *P. agglomerans* UADEC20, glycerol-3-phosphate, phosphate, and gluconate were obtained, which are related to the phosphate solubilization pathway.

#### 3.4.2. Enzymes Involved in the Biosynthesis, Transport, and Secretion of Exopolysaccharides (EPSs) from the *P. agglomerans* Genome

We explored the identification of enzymes involved in EPS biosynthesis, transport, and secretion in the *P. agglomerans* UADEC20 genome (Table 4). Twenty-three CDSs of enzymes involved in exopolysaccharide biosynthesis were identified, particularly in the subcategory of central carbohydrate metabolism/pentose phosphate pathway and extracellular polysaccharide synthesis. The synthesis of polysaccharides requires the biosynthesis of precursor molecules from the central metabolism of carbohydrates: in this first group, we found transketolase (EC 2.2.1.1), ribose-phosphate pyrophosphokinase (EC 2.7.6.1), ribulose-phosphate 3-epimerase (EC 5.1.3.1), glucose-6-phosphate 1-dehydrogenase (EC 1.1.1.49), 6-phosphogluconate dehydrogenase, decarboxylating enzyme (EC 1.1.1.44), 6-phosphogluconolactonase (EC 3.1.1.31), xylulose-5-phosphate phosphoketolase (EC 4.1.2.9), fructose-6-phosphate phosphoketolase (EC 4.1.2.22), and transaldolase (EC 2.2.1.2). In the extracellular polysaccharide synthesis group, we found dTDP-4-dehydrorhamnose reductase (EC 1.1.1.133), dTDP-glucose 4,6-dehydratase (EC 4.2.1.46), dTDP-4-dehydrorhamnose 3,5-epimerase (EC 5.1.3.13), glucose-1-phosphate thymidylyltransferase (EC 2.7.7.24), UDP-glucose 4-epimerase (EC 5.1.3.2), dTDP-4-dehydrorhamnose reductase (EC 1.1.1.133), dTDP-glucose 4,6-dehydratase (EC 4.2.1.46), dTDP-4-dehydrorhamnose 3,5-epimerase (EC 5.1.3.13), and glucose-1-phosphate thymidylyltransferase (EC 2.7.7.24). For the next phase of EPS biosynthesis, glycosyltransfers (GTs) play an important role in transferring the sugar nucleotides UDP-Glc/UDP-Gal/UDP-GlA to a repeating unit linked to a lipid transporter associated with the inner membrane. Within the category, D-inositol 3-phosphate glycosyltransferase, putative glycosyltransferase EpsJ, putative glycosyltransferase EpsJ, peptidoglycan glycosyltransferase MrdB, and putative peptidoglycan glycosyltransferase FtsW were identified.

In the *P. agglomerans* genome, we found 30 enzymes that participate in the Wzx-/Wzy-dependent pathway and the ABC transporter-dependent pathway. Within the Wzy-/Wzx-dependent pathway, in the genome, we found the presence of the *wzx*C_1 and 2 gene that encodes the Wzx flippase, Wzy polymerase (*wzy*E gene), pyrrolidone-carboxylate peptidase (*pcp* gene), and tyrosine-protein kinase wzc (*wzc* gene) genes; all of these are important elements for the production and transport of complex polysaccharides. Likewise, enzymes involved in the set of liposaccharides (LPSs) were identified: lipopolysaccharide assembly protein A and B, lipopolysaccharide heptosyltransferase 1, core lipopolysaccharide biosynthesis protein RfaG, ACE polysaccharide chain length modulation protein, core lipopolysaccharide heptosyltransferase RfaQ, lipopolysaccharide export system ATP-binding protein LptB, lipopolysaccharide export system protein LptA and LptC, and permease protein LptF and LptG.

The *P. agglomerans* UADEC20 strain possesses 12 genes encoding ATP-binding cassette ABC transporters, which are a family of integral membrane proteins for the ATP-dependent transport of various substrates, including putative phospholipid ABC transporter-binding MlaD, MlaB, YadG, and YheS proteins; inner membrane amino acid permease ABC transporter protein YhdY; inner membrane permease ABC transporter proteins YejE, YejB, YjfF, YtfT, and YdcV; periplasmic junctional ABC transporter protein YtfQ; and putative phospholipid ABC transporter protein MlaE. Within the EPS secretion system that this strain may contain, we find genes related to the membrane fusion protein of the type I secretion system PrsE (*prs*E_1,*2*) and the protein of the type II F and E secretion system (*eps*F and *gsp*E).

### 3.5. Virulence Genes

Using BLAST searches against the VFDB database [32] and genome annotation, a total of 37 putative virulence factors were predicted in *P. agglomerans* UADEC20 (Table S2). The virulence factors identified are classified mainly in motility; however, *tag*H is identified as a gene associated with the type VI secretion system (T6SS) and *omp*A is identified in adhesion. Overall, the strain seems to have low virulence capability.

### 3.6. Prophages and Drug Resistance Genes

In the analysis of antimicrobial resistance-related elements, only sequences associated with efflux pumps and genes with point mutations linked to resistance were detected. This indicates the presence of limited intrinsic resistance mechanisms, and the absence of mechanisms acquired through horizontal gene transfer (Table S3).

The phage analysis identified sequences associated with prophages containing stress response proteins, replication mechanism proteins such as helicases, and phage lysis modules. However, no intact phages were found in the genome (Table S4).

## 4. Discussion

### 4.1. Phylogenetic Analysis and Comparative Genomics

The phylogenetic tree based on the 16S rRNA gene positioned FDAARGOS 1447 at the root (Figure 1). The comparative analysis with the FDAARGOS 1447 strain using Mauve (Figure 2) revealed substantial genetic overlap. The Venn diagram of the core genome showed that these strains share a total of 3875 genes (Figure 5). Notably, UADEC20 has 49 CDSs with critical functions, such as *par*A, *vir*B, *int*A_3, *hyb*G, and *hem*S; the *hyc*ADEG CDS of the *hyc* operon; and the *hyp*ABDEF CDS of the *hyp* operon (Table S1); these CDSs are important in anaerobic metabolism, providing the strain with the biochemical capabilities to thrive in the environment. CDS *vir*B has been characterized as a key regulator of genes located on the high-virulence plasmid (pINV) in the bacterial pathogen *Shigella flexneri*; it is unrelated to other transcriptional regulators and belongs to a family of proteins that function primarily in plasmid and chromosomes [35]. CDS *par*A, known to be essential for plasmid cleavage, ensures the proper distribution of newly replicated plasmids to daughter cells during cell division [36]. The *int*A_3 CDS is also present in several rhizobia species, possibly with different specificities; it is known that the *int*A_3 system can be combined with other site-specific systems with different specificities to facilitate bacterial genome engineering [37]. It is known that the *hem*S CDS is also found in the *Bartonella* genome and contains a gene-encoding *hem*S that may be involved in the release of iron from heme [38]. The *hyc*ADEG operon encodes the products necessary for the formation of phosphate hydrogenase (FHL) present in UADEC20. This operon has also been reported in *Escherichia coli*, and *hyp*ABCDEF is found in many bacteria and archaea [39] (particularly *hyp*A and *hyp*B), which are involved in the insertion of the nickel atom into the large subunit precursor [40,41,42]. Coding sequences (CDSs) such as *hyc*ADGE have also been reported in *Enterobacter* species in the production of hydrogen, due to the enzyme hydrogenase 3 (encoded by *hyc*ABCDEFGHI); it presents important activities of hydrogen uptake and synthesis [43]. In *E. coli*, there is substantial evidence indicating that hydrogenase 3 activity is essential to produce hydrogen [44]; it has also been found to increase hydrogen yield in *E. aerogenes hyc*E or *hyc*G, which must be overexpressed in this species [45].

### 4.2. Metabolic Pathways Related to Phosphate Solubilization

We identified 57 coding sequences (CDSs) involved in phosphate solubilization (hereafter PS) metabolism within the chromosome of the UADEC20 strain (Table 3). This number surpasses the 10–40 CDSs reported in some bacteria of the same species [23,46,47]. The presence of intracellular enzymes involved in other metabolic processes, such as the pentose phosphate pathway, was detected. Additionally, CDSs corresponding to glucosyltransferases, which catalyze glucose residue transfer, crucial for polysaccharide synthesis, were annotated. For instance, UDP-glucose/alpha-D-galactose-1-phosphate uridylyltransferase catalyzes a key step in galactose metabolism. These enzymes are located in the periplasmic membrane, facilitating glucose residue accumulation for polysaccharide synthesis [48,49,50].

PS-associated genes include the *pqq* and *gdh* genes [51,52], which were also identified; they are critical for glucuronic acid production and gluconic acid secretion, respectively [53]. It is known that even if a strain has enzymes such as glucose dehydrogenase without the *pqq* genes, bacteria are not able to use it [54] (Table 3). These processes solubilize insoluble phosphates, with the *pqq*BCDE genes encoding enzymes essential for pyrroloquinoline quinone (PQQ) biosynthesis [51,52]. The strain also contains 17 *phn* genes related to phosphonate transport and degradation, compared to 13 in the P5 strain [55]. Additionally, the high-affinity phosphate transport system in UADEC20 includes eight *pst* operon genes, surpassing the four found in P5 [55] (Shariati et al. 2017). The *gcd* genes, involved in gluconic acid biosynthesis, further support UADEC20’s enhanced phosphate solubilization capabilities [56,57].

In the analysis of the predictions of the metabolic pathways obtained from the *P. agglomerans* UADEC20 genome, related to the phosphate solubilization mechanism, the following were found: D-gluconate degradation (GLUCONSUPER-PWY), glucose and glucose-1-phosphate degradation (GLUCOSE1PMETAB-PWY), sedoheptulose bisphosphate bypass (PWY0-1517), glycerol-3-phosphate to cytochrome bo oxidase electron transfer (PWY0-1561), succinate to cytochrome bd oxidase electron transfer (PWY0-1353), glycerol-3-phosphate shuttle (PWY-6118), phosphate acquisition (PWY-6348), and phosphate ABC transporter (PI-ABC-TRANS). In relation to the succinate-to-cytochrome bd oxidase electron transfer (PWY0-1353) predicted in the UADEC20 strain, Korshunov et al. [58] found that the *E. coli* bacterium can completely block primary cytochrome bd oxidase in the presence of low micromolar concentrations of hydrogen sulfide; it is known that this bacterium requires respiration for growth, and in such circumstances, respiration is maintained by shunting electrons to cytochrome bd oxidase; the authors conclude that the sulfur resistance of this enzyme may be critical to its role in bacteria.

### 4.3. Biosynthesis, Transport, and Secretion of Exopolysaccharides (EPSs)

The ability to synthesize and secrete polysaccharides is a notable property of PGPRs such as *Pantoea* [59]. These EPSs contribute to soil stability, water retention, and plant stress tolerance. The production of EPSs aids in protecting roots against phytopathogens and improving soil aeration and structure [59,60]. As mentioned earlier, the strain UADEC20 contains enzymes involved in the pentose phosphate pathway, which leads to polysaccharide synthesis (Table 4) [48,49,50]. These EPSs also contribute to maintaining the water film required for photosynthetic activity and plant growth, improving the process of soil aeration and infiltration, and covering and protecting roots against attack by phytopathogens. Under salt stress conditions, EPSs chelate cations available in the root zone, thus contributing to reducing the salinity of the rhizosphere. Bacterial EPSs under water stress conditions in the soil are responsible for limiting the desiccation of the medium. In the case of floods or heavy rain, EPSs help avoid the dispersion of clayey soils [60].

The Wzy-/Wzx-dependent pathway and ABC transporter systems for EPS transport are present in UADEC20. In the Wzy-/Wzx-dependent pathway, C55-anchored repeat units on the inner membrane are polymerized in the periplasm and exported to the cell surface [61,62,63]. Similarly, the ABC transporter system facilitates EPS export through a coordinated mechanism involving glycosyltransferases and transport proteins [64,65]. Enzymes for dTDP-rhamnose synthesis, a precursor for rhamnose found in lipopolysaccharides, capsular polysaccharides, and EPSs, were also identified [66].

In Gram-negative bacteria, six protein secretion systems have been identified to date, designated types I to VI, which differ greatly in composition and mechanism of action [67]. Analysis of the *P. agglomerans* genome indicates that it possesses type I and II EPS secretion systems [68]. The type I secretion system (T1SS) is responsible for transporting different molecules, mainly ions, carbohydrates, unfolded proteins, and RTX-type toxins [69,70,71]. The system is composed of an ABC transporter located on the inner membrane, a periplasmic tunneling protein, and a porin-like protein located on the outer membrane [71,72].

The type II secretion system (T2SS) depends on the Sec and Tat systems. These systems initiate secretion into the periplasmic space, which lies between the inner and outer membranes. Once in the periplasmic space, the transported molecule is secreted by the external part of the system that forms an exit channel. This external component is accompanied by another internal protein (chaperone) that ensures its stability by preventing it from degrading while it performs its job. The secreted molecules are mainly enzymes involved in degrading other membranes, so this system enhances bacterial competitiveness and pathogenesis capabilities [73,74,75,76].

In this study, we located enzymes involved in the synthesis of the capsule and extracellular polysaccharides, such as dTDP-4-dehydrorhamnose reductase. Further studies are needed to determine whether these LPSs can exhibit a bioactive effect. Polysaccharide synthesis and excretion properties are important for agricultural applications since these compounds are related to important phenomena such as biofilm formation in the rhizosphere, water and nutrient retention, osmotic stress protection, and better soil structure, and they also act as pathogen biocontrol effectors since their presence can activate defense mechanisms in the plants and protect the rhizome surface from pathogen colonization.

### 4.4. Virulence Genes and Antibiotic Resistance

The genomic analysis of UADEC20 showed the presence of 37 virulence genes. However, only a limited number of gene families associated with virulence factors were detected, e.g., those related to the type VI secretion system (T6SS), adhesion, and motility (Table S2). Among the identified virulence genes, the *tag*H gene encodes proteins that contain the *lpr*I lysozyme inhibitor domain [77], and the *omp*A gene codes for a membrane protein that contributes to the adhesion and evasion of host defenses [78]. Motility-associated genes play roles in biofilm formation, adhesion, and virulence factor secretion [79]. This indicates that UADEC20 exhibits very low virulence potential towards other organisms.

Similarly, only a few genes associated with intrinsic antibiotic resistance were identified, suggesting that the strain has limited resistance and virtually no capacity for transferring resistance genes to other strains. Despite these virulence-related genes, UADEC20 has low overall virulence potential and limited antibiotic resistance, justifying the further study of the strain to enhance its suitability for agricultural applications.

## 5. Conclusions

The strain of *Pantoea agglomerans* UADEC20 was identified and isolated from the rhizosphere of alfalfa (*Medicago sativa*) cultivated in northeastern Mexico. Key genes were identified in this study, which code for the biosynthesis, transport, and secretion of exopolysaccharides, as well as for phosphate solubilization, resistance to abiotic stresses (such as water scarcity and drought), and effective rhizosphere establishment. These features suggest significant potential for agricultural applications in Mexico. Notably, phylogenetic analyses and pangenome comparisons revealed that this strain exhibits a distinct evolutionary lineage compared to other species within the genus *Pantoea.*

Genomic analysis of UADEC20 indicated a comprehensive set of genes supporting phosphate solubilization, EPS synthesis, and secretion. Its low virulence and antibiotic resistance levels further justify its potential as a plant growth-promoting agent. Nonetheless, functional assays are necessary to validate its agricultural utility and determine its efficacy under field conditions. This finding highlights the potential biotechnological benefits associated with the unique genetic background of this strain.

## Figures and Tables

**Table 1:** Summary of *P. agglomerans* UADEC20 genome.

Replicon	No. of Illumina Reads	Reads After Clipping QC > 30%	Length Size (bp)	No. of Scaffolds	Scaffolds *N*_50_ (bp)	Maximum Length of Scaffolds	G+C Content (%)
Chromosome	20,949,701	20,873,634	4,203,428	13	209,743	1,523,742	54.94
Plasmid 1	-	-	543,479	1	-	-	53.6
Plasmid 2	-	-	174,380	1	-	-	52.0

%Q30 = The percentage of bases with a quality score of 30 or higher; %GC = percent content of Guanines and Cytosines.

**Table 2:** The genomes of *Pantoea agglomerans* strains in comparative genome analysis.

Organism	Genome Size (Mb)	GC%	No. of rRNA	No. of tRNA	Scaffolds	No. of Genes	No. of Proteins	GenBank Accession No.
*P. agglomerans* UADEC20	4.20343	54.9	22	99	13	4511	3843	GCA_046352745.1
*P. agglomerans* 299R	4.58148	54.3	27	63	109	4267	4157	GCA_000330765.1
*P. agglomerans* FDAARGOS 1447	3.99969	55.5	5	69	3	4361	4204	GCF_019048385.1
*P. agglomerans* Tx10	4.85699	55.1	48	142	22	4627	4500	GCA_000475055.1
*P. agglomerans* IG1	4.82958	55.0	2	63	18	4443	4341	GCA_000241285.2
*P. agglomerans* 190	5.00257	55.1	24	77	5	4878	4778	GCA_000731125.1
*P. agglomerans* P5	5.16726	55.4	7	63	150	4745	4674	GCA_002157425.2

**Table 3:** CDSs related to phosphate solubilization found in the annotated genome of *P. agglomerans* UADEC20. Functions were validated on Uniprot (https://www.uniprot.org/ [4 April 2024]).

No.	Gene	Protein	Locus Tag
1	*app*A	Oligopeptide-binding protein AppA	NDPIJCJM_00337
2	*gcd_*1	Quinoprotein glucose dehydrogenase	NDPIJCJM_01104
3	*gcd_*2	Quinoprotein glucose dehydrogenase	NDPIJCJM_02657
4	*gdh*l	Glucose dehydrogenase, PQQ-dependent (EC 1.1.5.2)	NDPIJCJM_02770
5	*glk*	Glucokinase	NDPIJCJM_00198
6	*gnt*K	Thermoresistant gluconokinase	NDPIJCJM_02222
7	*gnt*R	HTH-type transcriptional regulator GntR	NDPIJCJM_02223
8	*phn*C	Phosphate-import ATP-binding protein PhnC	NDPIJCJM_00558
9	*phn*D	Phosphate-import protein PhnD	NDPIJCJM_00559
10	*phn*E_1	Phosphate-import permease protein PhnE	NDPIJCJM_00560
11	*phn*E_2	Phosphate-import permease protein PhnE	NDPIJCJM_00561
12	*phn*F	Putative transcriptional regulator PhnF	NDPIJCJM_00548
13	*phn*G	Alpha-D-ribose 1-methylphosphonate 5-triphosphate synthase subunit PhnG (EC 2.7.8.37)	NDPIJCJM_00549
14	*phn*H	Alpha-D-ribose 1-methylphosphonate 5-triphosphate synthase subunit PhnH (EC 2.7.8.37)	NDPIJCJM_00550
15	*phn*I	Alpha-D-ribose 1-methylphosphonate 5-triphosphate synthase subunit PhnI (EC 2.7.8.37)	NDPIJCJM_00551
16	*phn*J	Alpha-D-ribose 1-methylphosphonate 5-phosphate C-P lyase	NDPIJCJM_00552
17	*phn*K	Putative phosphonates utilization ATP-binding protein PhnK	NDPIJCJM_00553
18	*phn*L	Alpha-D-ribose 1-methylphosphonate 5-triphosphate synthase subunit PhnL	NDPIJCJM_00554
19	*phn*M	Alpha-D-ribose 1-methylphosphonate 5-triphosphate diphosphatase	NDPIJCJM_00555
20	*phn*N	Ribose 1,5-bisphosphate phosphokinase PhnN	NDPIJCJM_00556
21	*phn*O	Aminoalkylphosphonate N-acetyltransferase	NDPIJCJM_01860
22	*phn*P	Phosphoribosyl 1,2-cyclic phosphate phosphodiesterase	NDPIJCJM_00557
23	*phn*V	Putative 2-aminoethylphosphonate transport system permease protein PhnV	NDPIJCJM_02365
24	*pho*B	Phosphate regulon transcriptional regulatory protein PhoB (SphR)	NDPIJCJM_02028
25	*pho*H	Phosphate starvation-inducible protein PhoH, predicted ATPase	NDPIJCJM_00650
26	*pho*P	Transcriptional regulatory protein PhoP	NDPIJCJM_03210
27	*pho*R	Phosphate regulon sensor protein PhoR (SphS) (EC 2.7.13.3)	NDPIJCJM_02027
28	*pho*U	Phosphate-specific transport system accessory protein PhoU	NDPIJCJM_02471
29	*phy*	Phytase	NDPIJCJM_00083
30	*pit*A_1	Low-affinity inorganic phosphate transporter 1	NDPIJCJM_01727
31	*pit*A_2	Low-affinity inorganic phosphate transporter 1	NDPIJCJM_02277
32	*ppa*	Inorganic pyrophosphatase	NDPIJCJM_03544
33	*ppk*	T6SS Serine/threonine protein kinase (EC 2.7.11.1) PpkA	NDPIJCJM_00117
34	*ppx*	Exopolyphosphatase	NDPIJCJM_00116
35	*pqq*B	Coenzyme PQQ synthesis protein B	NDPIJCJM_01165
36	*pqq*C	Pyrroloquinoline-quinone synthase	NDPIJCJM_01164
37	*pqq*D	Coenzyme PQQ synthesis protein D	NDPIJCJM_01163
38	*pqq*E	Coenzyme PQQ synthesis protein E	NDPIJCJM_01166
39	*psi*F	Phosphate starvation-inducible protein PsiF	NDPIJCJM_02051
40	*pst*A_1	Phosphate transport system permease protein PstA	NDPIJCJM_00119
41	*pst*A_2	Phosphate transport system permease protein PstA	NDPIJCJM_00119
42	*pst*B	Phosphate import ATP-binding protein PstB	NDPIJCJM_02470
43	*pst*C	Phosphate transport system permease protein PstC	NDPIJCJM_02468
44	*pst*S_1	Phosphate-binding protein PstS	NDPIJCJM_02026
45	*pst*S_2	Phosphate-binding protein PstS	NDPIJCJM_02026
46	*ugp*A	sn-glycerol-3-phosphate transport system permease protein UgpA	NDPIJCJM_02231
47	*ugp*B	sn-glycerol-3-phosphate-binding periplasmic protein UgpB	NDPIJCJM_02232
48	*ugp*C	sn-glycerol-3-phosphate import ATP-binding protein UgpC	NDPIJCJM_02229
49	*ugp*Q	Glycerophosphodiester phosphodiesterase, cytoplasmic	NDPIJCJM_02228
50	*ush*A	Protein UshA	NDPIJCJM_01942
51	*zwf*	Glucose-6-phosphate 1-dehydrogenase	NDPIJCJM_00714
52	*ptx*S	2-ketogluconate utilization repressor *ptxS*	NDPIJCJM_02300
53	*glp*T	Glycerol-3-phosphate transporter	NDPIJCJM_02625
54	*uhp*T	Hexose-6- phosphate:phosphate antiporter	NDPIJCJM_02853
55	*phn*R	Putative transcriptional regulator of 2-aminoethylphosphonate degradation operons	NDPIJCJM_02369
56	*pst*C1	Phosphate transport system permease protein PstC 1	NDPIJCJM_00118
57	*pst*B3	Phosphate import ATP-binding protein PstB 3	NDPIJCJM_00120

**Table 4:** Genes and proteins related to the biosynthesis, transport, and secretion of exopolysaccharides identified in the genome of *P. agglomerans* UADEC20.

No.	Gene	Protein	Locus Tag
Biosynthesis of exopolysaccharides			
1	*tkt*B	Transketolase 2	NDPIJCJM_00157
2	*cbb*T	Transketolase 2	NDPIJCJM_03242
3	*tkt*A	Transketolase 1	NDPIJCJM_03750
4	*prs*	Ribose-phosphate pyrophosphokinase	NDPIJCJM_01374
5	*rpe*	Ribulose-phosphate 3-epimerase	NDPIJCJM_02168
6	*zwf*	Glucose-6-phosphate 1-dehydrogenase	NDPIJCJM_00714
7	*gnd*	6-phosphogluconate dehydrogenase, decarboxylating	NDPIJCJM_00429
8	*pgl_*1	6-phosphogluconolactonase	NDPIJCJM_00577
9	*tal*	Transaldolase	NDPIJCJM_00158
10	*rfb*D	dTDP-4-dehydrorhamnose reductase	NDPIJCJM_00418
11	*rff*G	dTDP-glucose 4,6-dehydratase 2	NDPIJCJM_02591
12	*rfb*B	dTDP-glucose 4,6-dehydratase	NDPIJCJM_00417
13	*rfb*C	dTDP-4-dehydrorhamnose 3,5-epimerase	NDPIJCJM_00420
14	*rff*H	Glucose-1-phosphate thymidylyltransferase	NDPIJCJM_02592
15	*gal*E	UDP-glucose 4-epimerase	NDPIJCJM_00416
16	*rfb*D	dTDP-4-dehydrorhamnose reductase	NDPIJCJM_00418
17	*rff*G	dTDP-glucose 4,6-dehydratase 2	NDPIJCJM_02591
18	*rfb*C	dTDP-4-dehydrorhamnose 3,5-epimerase	NDPIJCJM_00420
19	*msh*A	D-inositol 3-phosphate glycosyltransferase	NDPIJCJM_00425
20	*epsJ*_1	Putative glycosyltransferase EpsJ	NDPIJCJM_01496
21	*epsJ*_2	Putative glycosyltransferase EpsJ	NDPIJCJM_01584
22	*mrd*B	Peptidoglycan glycosyltransferase MrdB	NDPIJCJM_01847
23	*fts*W	Putative peptidoglycan glycosyltransferase FtsW	NDPIJCJM_02969
Polysaccharide transport and export system			
24	*wzx*C_1	Lipopolysaccharide biosynthesis protein WzxC	NDPIJCJM_00414
25	*wzx*C_2	Lipopolysaccharide biosynthesis protein WzxC	NDPIJCJM_01530
26	*wzy*E	Putative ECA polymerase	NDPIJCJM_02597
27	*pcp*	Pyrrolidone-carboxylate peptidase	NDPIJCJM_01797
28	*wzc*	Tyrosine-protein kinase Wzc	NDPIJCJM_00404
29	*lap*A	Lipopolysaccharide assembly protein A	NDPIJCJM_00864
30	*lap*B	Lipopolysaccharide assembly protein B	NDPIJCJM_00865
31	*rfa*C	Lipopolysaccharide heptosyltransferase 1	NDPIJCJM_01493
32	*rfa*G	Lipopolysaccharide core biosynthesis protein RfaG	NDPIJCJM_01498
33	*wzz*E	ECA polysaccharide chain length modulation protein	NDPIJCJM_02588
34	*rfa*Q_2	Lipopolysaccharide core heptosyltransferase RfaQ	NDPIJCJM_03222
35	*rfa*Q_3	Lipopolysaccharide core heptosyltransferase RfaQ	NDPIJCJM_03224
36	*lpt*B_1	Lipopolysaccharide export system ATP-binding protein LptB	NDPIJCJM_02234
37	*lpt*B_2	Lipopolysaccharide export system ATP-binding protein LptB	NDPIJCJM_02719
38	*lpt*A	Lipopolysaccharide export system protein LptA	NDPIJCJM_02720
39	*lpt*C	Lipopolysaccharide export system protein LptC	NDPIJCJM_02721
40	*lpt*F	Lipopolysaccharide export system permease protein LptF	NDPIJCJM_02803
41	*lpt*G	Lipopolysaccharide export system permease protein LptG	NDPIJCJM_02804
42	*lpt*B_3	Lipopolysaccharide export system ATP-binding protein LptB	NDPIJCJM_02899
43	*mla*D	Putative phospholipid ABC transporter-binding protein MlaD	NDPIJCJM_02727
44	*mla*B	Putative phospholipid ABC transporter-binding protein MlaB	NDPIJCJM_02729
45	*yad*G	Putative ABC transporter ATP-binding protein YadG	NDPIJCJM_03019
46	*yhd*Y	Inner membrane amino-acid ABC transporter permease protein YhdY	NDPIJCJM_03327
47	*yej*E	Inner membrane ABC transporter permease protein YejE	NDPIJCJM_00335
48	*yej*B	Inner membrane ABC transporter permease protein YejB	NDPIJCJM_00336
49	*yjf*F	Inner membrane ABC transporter permease protein YjfF	NDPIJCJM_01202
50	*ytf*T	Inner membrane ABC transporter permease protein YtfT	NDPIJCJM_01203
51	*ytf*Q	ABC transporter periplasmic-binding protein YtfQ	NDPIJCJM_01205
52	*ydc*V	Inner membrane ABC transporter permease protein YdcV	NDPIJCJM_01647
53	*yhe*S	Putative ABC transporter ATP-binding protein YheS	NDPIJCJM_02143
54	*mla*E	Putative phospholipid ABC transporter permease protein MlaE	NDPIJCJM_02726
System secretion			
55	*prs*E_1	Type I secretion system membrane fusion protein PrsE	NDPIJCJM_00316
56	*prs*E_2	Type I secretion system membrane fusion protein PrsE	NDPIJCJM_02849
57	*eps*F	Type II secretion system protein F	NDPIJCJM_02984
58	*gsp*E	Putative type II secretion system protein E	NDPIJCJM_02985

## Data Availability

Nucleotide sequence accession numbers. This genome sequence was deposited in GenBank under the accession numbers CP125809, CP125810, and CP125811 with BioProject number PRJNA955887.

## Supplementary Materials

The following supporting information can be downloaded at https://www.mdpi.com/article/10.3390/cimb47010056/s1.
