# Supplementary material for: Insights from a Genome-Wide Study of Pantoea agglomerans UADEC20: A Promising Strain for Phosphate Solubilization and Exopolysaccharides Production
Source: Curr Issues Mol Biol. 2025 Jan 16;47(1):56. doi: 10.3390/cimb47010056 (PMC11763638; doi:10.3390/cimb47010056)
Supplement: Supplementary file 1 [file cimb-47-00056-s001.zip › cimb-3397107-supplementary.pdf]

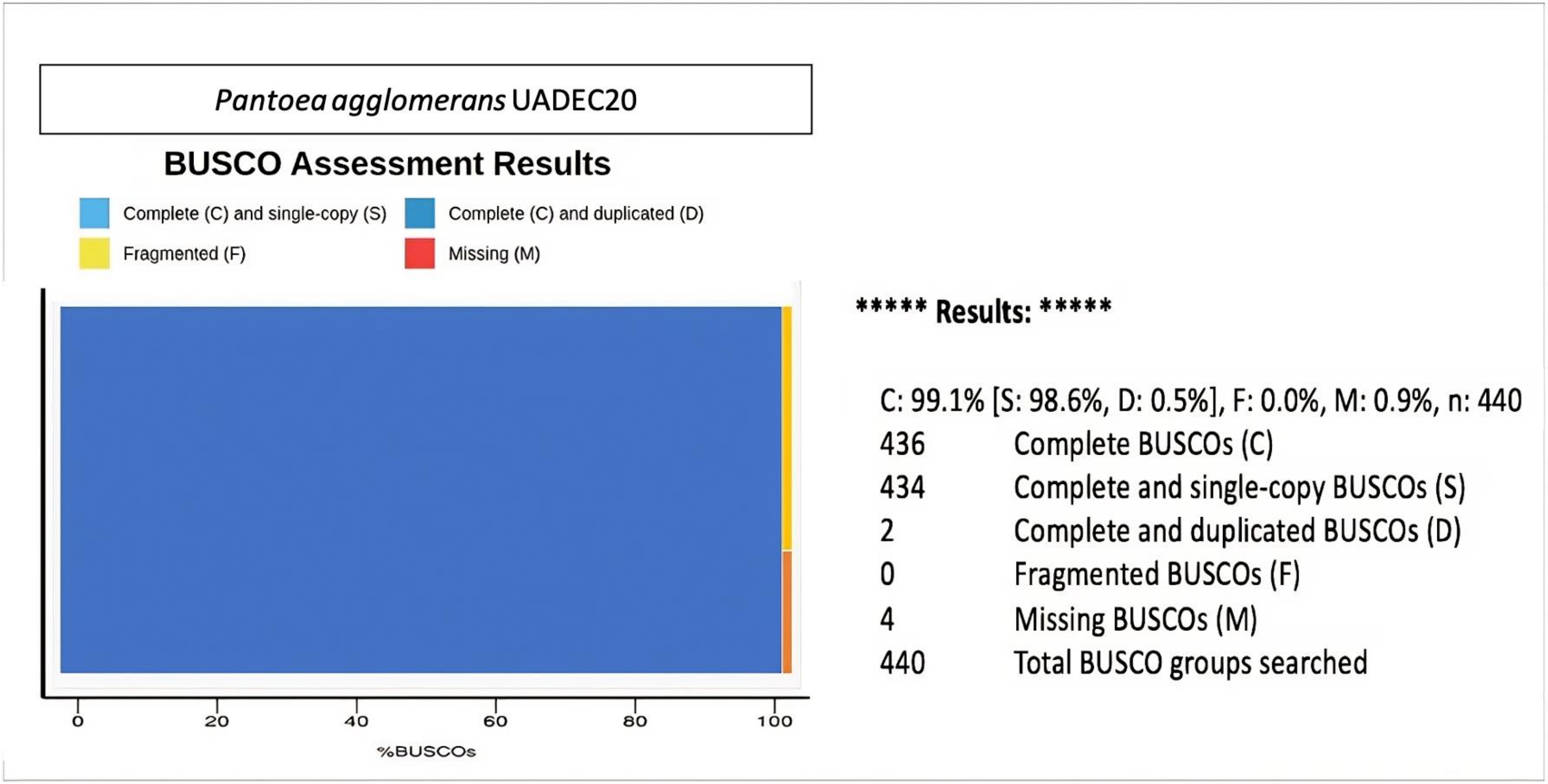

Figure S1: Evaluation of the integrity of the genome assembly and annotation of *P. agglomerans* UADEC20 with BUSCO tool [18]. The percentage of single-copy complete orthologs (light blue), fragmented or incomplete orthologs (yellow), complete and duplicate orthologs (blue), and missing orthologs (red) are shown.

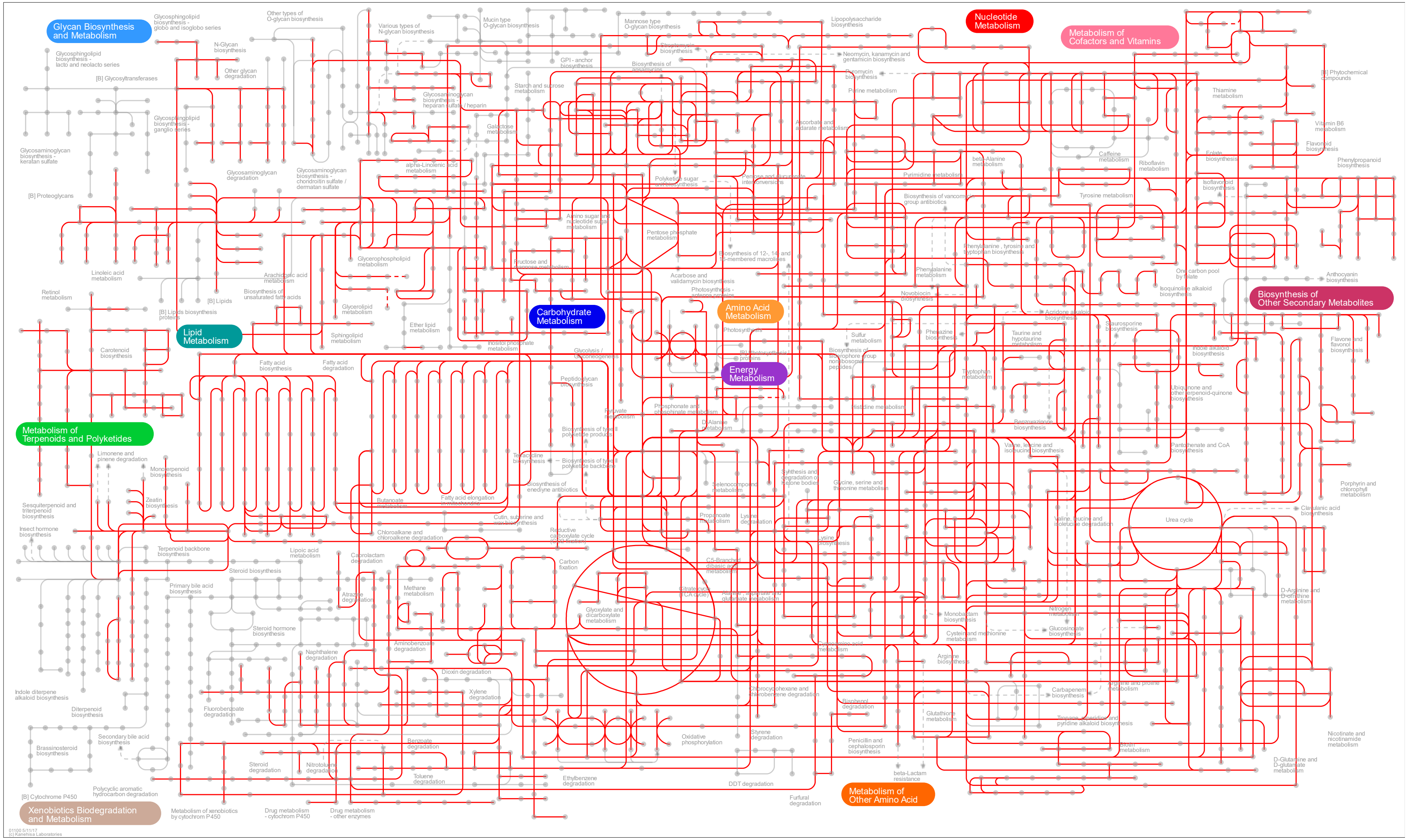

Figure S2: Visualization of the reduced metabolic pathways identified in *P. agglomerans*. Red lines represent reactions catalyzed by enzymes identified after functional annotation (annotation and KOs assigned with Prokka [23]) and visualized on the KEGG's microbial metabolism map using iPath v3.0 tool.

Table S1. Coding sequences (CDS) unique to *P. agglomerans* UADEC20 identified by comparative genomics to *P. agglomerans* FDAARGOS 1447.

No.	Gene	Product	Locus tag
1	*par*A	Plasmid partition protein A	NDPIJCJM_03381
2	*vir*B	Virulence regulon transcriptional activator VirB	NDPIJCJM_03382
3	*fim*D_3	Outer membrane usher protein FimD	NDPIJCJM_03390
4	*int*A_3	Prophage integrase IntA	NDPIJCJM_03604
5	*hem*S	Hemin transport protein HemS	NDPIJCJM_03717
6	*hmu*T	Hemin-binding periplasmic protein HmuT	NDPIJCJM_03718
7	*cod*B_2	Cytosine permease	NDPIJCJM_02181
8	*int*Q	Putative defective protein IntQ	NDPIJCJM_00919
9	*dna*T_1	Primosomal protein 1	NDPIJCJM_00930
10	*xer*C_1	Tyrosine recombinase XerC	NDPIJCJM_00708
11	*rha*S	HTH-type transcriptional activator RhaS	NDPIJCJM_01032
12	*codAch*2	Pterin deaminase	NDPIJCJM_00492
13	*crn*A	Creatinine amidohydrolase	NDPIJCJM_00490
14	*mac*B	Macrolide export ATP-binding/permease protein MacB	NDPIJCJM_03400
15	*btu*D	Vitamin B12 import ATP-binding protein BtuD	NDPIJCJM_01302
16	*yed*K	Putative SOS response-associated peptidase YedK	NDPIJCJM_03472
17	*pur*H	Bifunctional purine biosynthesis protein PurH	NDPIJCJM_03490
18	*gpp*A_1	Guanosine-5'-triphosphate,3'-diphosphate pyrophosphatase	NDPIJCJM_02583
19	*hyp*E	Carbamoyl dehydratase HypE	NDPIJCJM_01142
20	*hyp*D	Hydrogenase maturation factor HypD	NDPIJCJM_01143
21	*hyb*G	Hydrogenase maturation factor HybG	NDPIJCJM_01144
22	*hyp*B	Hydrogenase maturation factor HypB	NDPIJCJM_01145
23	*hyp*A	Hydrogenase maturation factor HypA	NDPIJCJM_01146
24	*sac*A	Sucrose-6-phosphate hydrolase	NDPIJCJM_00541
25	*dau*A_1	C4-dicarboxylic acid transporter DauA	NDPIJCJM_00536
26	*rib*Y	Riboflavin-binding protein RibY	NDPIJCJM_00496
27	*cyn*R_2	HTH-type transcriptional regulator CynR	NDPIJCJM_00181
28	*rec*D2	ATP-dependent RecD-like DNA helicase	NDPIJCJM_03611
29	*rec*F_1	DNA replication and repair protein RecF	NDPIJCJM_01633
30	*rep*	ATP-dependent DNA helicase Rep	NDPIJCJM_02582
31	*tra*C	Protein TraC	NDPIJCJM_03436
32	*ccd*A	Antitoxin CcdA	NDPIJCJM_03475
33	*ccd*B	Toxin CcdB	NDPIJCJM_03476
34	*fdh*F	Formate dehydrogenase H	NDPIJCJM_01157
35	*hyp*F	Carbamoyltransferase HypF	NDPIJCJM_01158
36	*fhl*A	Formate hydrogenlyase transcriptional activator	NDPIJCJM_01141

37	*ula*A	Ascorbate-specific PTS system EIIC component	NDPIJCJM_01138
38	*pfk*A_1	ATP-dependent 6-phosphofructokinase	NDPIJCJM_01140
39	*csc*B	Sucrose permease	NDPIJCJM_00540
40	*rbs*R_1	Ribose operon repressor	NDPIJCJM_00539
41	*oph*A1	Phthalate dioxygenase reductase	NDPIJCJM_00493
42	*msc*M_1	Miniconductance mechanosensitive channel MscM	NDPIJCJM_01721
43	*rhs*C_1	Protein RhsC	NDPIJCJM_01413
44	*rhs*C_2	Putative deoxyribonuclease RhsC	NDPIJCJM_01426
45	*nrt*A	Nitrate transport protein NrtA	NDPIJCJM_01353
46	*hyc*A	Formate hydrogenlyase regulatory protein hycA	NDPIJCJM_01147
47	*hyc*D	Formate hydrogenlyase subunit 4	NDPIJCJM_01150
48	*hyc*E	Formate hydrogenlyase subunit 5	NDPIJCJM_01151
49	*hyc*G	Formate hydrogenlyase subunit 7	NDPIJCJM_01153

Table S2. Virulence factors of *P. agglomerans* UADEC20.

No.	Virulence gene	Virulence factor	Product	Locus tag
1	*fli*C	Motility	Flagellin	DDDKMLIA_03153
2	*fli*D	Motility	Flagellar hook-associated protein 2	DDDKMLIA_01021
3	*fli*E	Motility	Flagellar hook-basal body complex protein FliE	DDDKMLIA_01004
4	*fli*F	Motility	Flagellar M-ring protein	DDDKMLIA_01003
5	*fli*G	Motility	Flagellar motor switch protein FliG	DDDKMLIA_01002
6	*fli*H	Motility	Flagellar assembly protein FliH	DDDKMLIA_01001
7	*fli*I	Motility	Flagellum-specific ATP synthase	DDDKMLIA_01000
8	*fli*J	Motility	Flagellar FliJ protein	DDDKMLIA_00999
9	*fli*K	Motility	Flagellar hook-length control protein	DDDKMLIA_00998
10	*fli*M	Motility	Flagellar motor switch protein FliM	DDDKMLIA_00996
11	*fli*N	Motility	Flagellar motor switch protein FliN	DDDKMLIA_00995
12	*fli*O	Motility	Flagellar protein FliO	DDDKMLIA_00994
13	*fli*S	Motility	Flagellar secretion chaperone FliS	DDDKMLIA_01020
14	*fli*T	Motility	Flagellar protein FliT	DDDKMLIA_01019
15	*fli*Z	Motility	Regulator of sigma S factor FliZ	DDDKMLIA_01028
16	*flh*A	Motility	Flagellar biosynthesis protein FlhA	DDDKMLIA_01064
17	*flh*C	Motility	Flagellar transcriptional regulator FlhC	DDDKMLIA_01051
18	*flh*D	Motility	Flagellar transcriptional regulator FlhD	DDDKMLIA_01050
19	*flh*E	Motility	Flagellar protein FlhE	DDDKMLIA_01065
20	*flg*A	Motility	Flagella basal body P-ring formation protein FlgA	DDDKMLIA_04222
21	*flg*B	Motility	Flagellar basal body rod protein FlgB	DDDKMLIA_04223

22	*flg*C	Motility	Flagellar basal-body rod protein FlgC	DDDKMLIA_04224
23	*flg*D	Motility	Basal-body rod modification protein FlgD	DDDKMLIA_04225
24	*flg*E	Motility	Flagellar hook protein FlgE	DDDKMLIA_04226
25	*flg*F	Motility	Flagellar basal-body rod protein FlgF	DDDKMLIA_04227
26	*flg*G	Motility	Flagellar basal-body rod protein FlgG	DDDKMLIA_04228
27	*flg*H	Motility	Flagellar L-ring protein	DDDKMLIA_04229
28	*flg*I	Motility	Flagellar P-ring protein	DDDKMLIA_04230
29	*flg*J	Motility	Peptidoglycan hydrolase FlgJ	DDDKMLIA_04231
30	*flg*K	Motility	Flagellar hook-associated protein 1	DDDKMLIA_04232
31	*flg*L	Motility	Flagellar hook-associated protein 3	DDDKMLIA_04233
32	*flg*N	Motility	Flagella synthesis protein FlgN	DDDKMLIA_04220
33	*omp*A	Adhesion	Outer membrane protein A	DDDKMLIA_02476
34	*tag*H	Secretion system	Teichoic acids export ATP-binding protein TagH	DDDKMLIA_00813
35	*vir*B	Transcription	Virulence regulon transcriptional activator VirB	IKFMONDD_00021
36	*pho*Q	Phosphorelay signal transduction system	Virulence sensor histidine kinase PhoQ	NDPIJCJM_03209
37	*pho*P	Phosphorelay signal transduction system	Virulence transcriptional regulatory protein PhoP	NDPIJCJM_03210

Table S3. Antimicrobial resistance genes identified in *P. agglomerans* UADEC20 genome using the CARD database.

Gene	Product	ARO id	CARD Organism	Mechanism class	Resistance to	Score	Ident %
*bep*E	Efflux pump membrane transporter BepE	ARO:3000777	*Acinetobacter baumannii* AYE	antibiotic efflux	fluoroquinolone; tetracycline	1205.3	60.8
*msb*A	ATP-binding lipopolysaccharide transport protein	ARO:3003950	*Escherichia coli* str. K-12 substr. MG1655	antibiotic efflux	metronidazole	1063.1	87.29
*acr*B	multidrug efflux pump RND permease AcrB	ARO:3000777	*Acinetobacter baumannii* AYE	antibiotic efflux	fluoroquinolone; tetracycline	763.1	41.51
crp	DNA-binding transcriptional dual regulator CRP	ARO:3000518	*Escherichia coli* str. K-12 substr. W3110	antibiotic efflux	norfloxacino; oxacillin; cloxacilline; erythromycin	431.8	98.57
*mpr*A	DNA-binding transcriptional repressor MprA	ARO:3000516	*Escherichia coli* str. K-12 substr. MG1655	antibiotic efflux	nalidixic acid	287	80.12
*gyr*B	DNA gyrase subunit B	ARO:3003306	*Morganella*	antibiotic target alteration	fluoroquinolone; perfloxacin; sparfloxacin; grepafloxacin; trovafloxacin; ofloxacin; norfloxacin; nalidixic acid; lomefloxacin; gatifloxacin; moxifloxacin; levofloxacin; ciprofloxacin; enoxacin	1334.7	80.85
*uhp*T	hexose-6-phosphate:phosphate antiporter	ARO:3003890	*Escherichia coli* str. K-12 substr. MC4100	antibiotic target alteration	Fosfomycin	856.3	92.87
*fts*I	peptidoglycan DD-transpeptidase FtsI	ARO:3004446	*Haemophilus influenzae* Rd KW20	antibiotic target alteration	cephamycin; cephalosporin; beta-lactam antibiotic	592.4	53.19

Table S4. Bacteriophage sequences detected within *P. agglomerans* UADEC20 genome.

Category	Subcategory	Subsystem	Role	Features
Stress Response	Stress Response - no subcategory	Phage shock protein (psp) operon	Phage shock protein B	fig|6666666.968136.peg.2192
Stress Response	Stress Response - no subcategory	Phage shock protein (psp) operon	Psp operon transcriptional activator	fig|6666666.968136.peg.2190
Stress Response	Stress Response - no subcategory	Phage shock protein (psp) operon	Phage shock protein C	fig|6666666.968136.peg.2193
Stress Response	Stress Response - no subcategory	Phage shock protein (psp) operon	Phage shock protein A	fig|6666666.968136.peg.2191
Stress Response	Stress Response - no subcategory	Phage shock protein (psp) operon	Phage shock protein D	fig|6666666.968136.peg.2194
"Phages, Prophages, Transposable elements, Plasmids"	"Phages, Prophages"	Phage baseplate proteins	Phage baseplate protein	fig|6666666.968136.peg.2263
"Phages, Prophages, Transposable elements, Plasmids"	"Phages, Prophages"	Phage replication	DNA polymerase III alpha subunit (EC 2.7.7.7)	fig|6666666.968136.peg.420
"Phages, Prophages, Transposable elements, Plasmids"	"Phages, Prophages"	Phage replication	"DNA helicase (EC 3.6.1.-), phage-associated"	fig|6666666.968136.peg.179
"Phages, Prophages, Transposable elements, Plasmids"	"Phages, Prophages"	Phage lysis modules	Phage outer membrane lytic protein Rz	fig|6666666.968136.peg.2237
"Phages, Prophages, Transposable elements, Plasmids"	"Phages, Prophages"	Phage lysis modules	Phage holin	fig|6666666.968136.peg.499
